# Previous institutionalization is associated with elevated functional connectivity between the nucleus accumbens and amygdala during aversive learning

**DOI:** 10.1016/j.dcn.2025.101617

**Published:** 2025-09-26

**Authors:** Benjamin M. Rosenberg, João F. Guassi Moreira, Adriana S. Méndez Leal, Natalie M. Saragosa-Harris, Elizabeth Gaines, Wesley J. Meredith, Clare F. McCann, Saché M. Coury, Yael Waizman, Emilia Ninova, Jennifer A. Silvers

**Affiliations:** a Neuroscience Department, Pomona College, Claremont, CA, USA; b Department of Psychology, University of Wisconsin, Madison, WI, USA; c Department of Psychology, University of California, Los Angeles (UCLA), Los Angeles, CA, USA; d Department of Psychology, University of Southern California, Los Angeles, CA, USA; e College of Social Work, Florida State University, Tallahassee, FL, USA

**Keywords:** Early Life Adversity, Institutionalization, Adolescence, Threat Learning, Functional Connectivity, Anxiety, Depression

## Abstract

Institutionalization is a profound form of early adversity that is associated with increased risk for internalizing disorders, which most commonly have their onset during adolescence. Developmental models emphasize how differences in childhood learning contribute to avoidance behaviors, a core pathway linking adversity to internalizing disorders. Yet, little empirical research has tested this theory. 43 previously institutionalized (PI; 12.1–22.8 years) and 47 comparison (9.9–22.9 years) youth completed an aversive learning task while undergoing fMRI. The task involved an escapable stimulus reinforced with an aversive sound (CS+_r_), the same stimulus without reinforcement (CS+_nr_), and an escapable stimulus that was never reinforced (CS-). Internalizing symptoms were measured using the parent-report Revised Child Anxiety and Depression Scales. Functional connectivity between the nucleus accumbens (NAcc) and amygdala was elevated among the PI versus comparison youth across stimuli (*p* = .036). Exploratory analyses found that NAcc-amygdala connectivity was elevated among the PI youth during early adolescence relative to late adolescence (*p* = .009). Institutionalization may impact neurodevelopment in ways that increase responsiveness of threat neurocircuitry across threatening and safe stimuli. Differences in NAcc-amygdala functional connectivity may attenuate with age following adoption.

## Highlights

• Institutionalization in orphanage care is a profound form of early life adversity.
• Nucleus accumbens and amygdala coordinate to differentiate threat versus safety.
• Previous institutionalization alters this neurocircuitry during adolescence.
• Institutionalization-related differences may attenuate with age following adoption.

## Graphical Abstract

**Figure:**
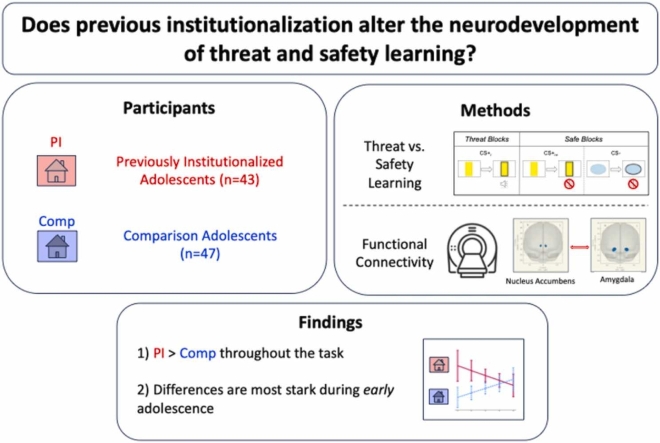

## 1 Introduction

### 1.1 Early adversity and risk for psychopathology

Experiences of early life adversity (ELA) are associated with heightened risk for developing psychological disorders (Green et al., 2010a, Kessler et al., 2010, Li et al., 2016, McLaughlin et al., 2010), especially during adolescence (McLaughlin et al., 2012). Rates of onset for internalizing disorders, such as anxiety and depression in particular, are higher during adolescence than any other point in the human lifespan (Kessler et al., 2007, Kessler et al., 2012, Merikangas et al., 2010, Paus et al., 2008, Powers and Casey, 2015, Rapee et al., 2019). One proposed mechanism linking ELA to internalizing symptoms is impaired associative learning (Hanson et al., 2017), particularly when ELA interferes with discrimination between threats versus safety (McLaughlin et al., 2020, McLaughlin and Lambert, 2017). Nonetheless, much remains unknown about the specific psychological and neural mechanisms linking ELA to internalizing psychopathology.

### 1.2 ELA and associative learning

Learning to effectively distinguish threat and safety is a central task in human development, and such learning is often altered by exposure to ELA (McLaughlin et al., 2019). Individuals exposed to ELA often encounter unpredictable environments and consequently exhibit poor discrimination learning during development (Ellis et al., 2022, Harhen and Bornstein, 2024), especially for threatening versus safe stimuli (Hartley et al., 2014, Jovanovic et al., 2022, McLaughlin et al., 2014, McLaughlin et al., 2015, McLaughlin et al., 2020; McLaughlin and Lambert, 2017), although there is some evidence that youth exposed to interpersonal trauma show intact safety learning (Kribakaran et al., 2024). ELA in the form of maltreatment (e.g., abuse, neglect, or institutionalization) during childhood may alter threat and safety learning by enhancing threat detection and reducing attention to contextual factors that signal safety (McLaughlin and Lambert, 2017) or through its association with blunted physiological responding to threatening stimuli (Klingelhöfer-Jens et al., 2025, Machlin et al., 2019, McLaughlin et al., 2015). Developmental models of anxiety disorders emphasize the importance of threat and safety discrimination, as ambiguity can increase reliance on behavioral strategies that perpetuate anxiety long-term (e.g., avoidance) (Waters and Craske, 2016). For example, overgeneralization of fear has been shown to moderate the association between childhood maltreatment and trait symptoms of anxiety and depression during emerging adulthood (Lange et al., 2019).

While numerous theoretical and empirical lines of inquiry have pointed to threat neurocircuitry as a mechanistic link between ELA exposure and altered threat and safety learning, less work has considered reward circuitry in this vein. This is surprising given that many forms of ELA exposure have been linked to deficits in reinforcement learning and reward processes (Gerin et al., 2017a, Gerin et al., 2017b, Guassi Moreira et al., 2022, Hanson et al., 2017, Lloyd et al., 2022, Loman et al., 2014, Pechtel and Pizzagalli, 2011). Moreover, reward processes are theorized to support central mechanisms of threat and safety discrimination, such as Pavlovian fear extinction (Rosenberg, Barnes-Horowitz, et al., 2024). For example, symptoms of anhedonia (i.e., low reward sensitivity, motivation, or pursuit) are associated with widespread patterns of unique brain activity during Pavlovian fear extinction (Rosenberg et al., 2023) and overgeneralization of fear to safe stimuli (Rosenberg et al., 2024).

### 1.3 ELA and neurodevelopment

Existing research suggests that ELA alters the structure and function of several brain regions during development, including the nucleus accumbens (NAcc), which is typically considered in relation to reward processes (Dillon et al., 2009, Fareri et al., 2017, Fareri and Tottenham, 2016, Goff et al., 2013a, Goff and Tottenham, 2015, Hanson et al., 2015, Mehta et al., 2010a, Tottenham and Galván, 2016), and the amygdala, which is typically examined in the context of threat processes (Cohen et al., 2013, DeCross et al., 2022, Fareri and Tottenham, 2016, Gee et al., 2013, Silvers et al., 2016, Tottenham et al., 2010, Tottenham et al., 2011, Tottenham and Galván, 2016). These alterations may not always be maladaptive. For example, accelerated neurodevelopment of threat circuitry following adversity exposure may help children meet immediate survival demands (Callaghan and Tottenham, 2016). Although accelerated neurodevelopment may be protective in the short-term, premature emphasis on emotional and associative learning may increase future vulnerability for anxiety disorders by interfering with discrimination of threat versus safety (Callaghan and Tottenham, 2016, McLaughlin et al., 2019). For example, individuals with more exposure to ELA show greater similarity in NAcc and amygdala representations of threatening and ambiguous stimuli (Saragosa-Harris et al., 2024). Additional research is needed to elucidate how alterations to emotional learning correspond with neurodevelopmental changes following ELA, as these mechanisms may present opportune targets for psychotherapy (McLaughlin, DeCross, et al., 2019).

### 1.4 Previous institutionalization

One unique and highly impactful form of ELA is institutionalization in orphanage care during early childhood, which significantly increases the risk of anxiety or depression symptoms during adolescence, even after early adoption (Gunnar and Bowen, 2021, Humphreys et al., 2015, Waizman et al., 2021). Previously institutionalized (PI) youth are especially unique in that they are exposed to a profound stressor (i.e., separation from caregiver) during early childhood but may be subsequently adopted into a highly enriched environment with supportive caregiving, thus limiting their experience of ELA to a discrete period. Prior studies have shown that institutionalization is associated with heightened resting-state functional connectivity between the amygdala and ventromedial prefrontal cortex (Herzberg et al., 2021), enhanced amygdala-mediated vigilance to threats (Silvers et al., 2017), and less amygdalar discrimination between images of their parent versus a stranger (Callaghan et al., 2019, Olsavsky et al., 2013). These studies collectively suggest that institutionalization may alter amygdalar mechanisms of threat and safety discrimination. Institutionalization is also associated with hyporesponsivity of the NAcc to rewards in adolescence (Goff et al., 2013b, Mehta et al., 2010b, Sheridan et al., 2018), coinciding with lower sensitivity to rewards during decision making (Guassi Moreira et al., 2022) and less sensation-seeking behavior (Loman et al., 2014). Given the profound links between institutionalization and alterations to psychological and neurodevelopmental processes relevant to anxiety and depression, PI youth present as an especially compelling group of individuals for further study.

To our knowledge, only one study (by our group) has evaluated PI youth during aversive learning, showing that children and adolescents with a history of institutionalization exhibited intact threat learning but responded to threats with 1) greater activation of the hippocampus, but not the amygdala, 2) greater functional connectivity between the hippocampus and ventromedial prefrontal cortex, and 3) greater functional connectivity between the amygdala and ventromedial prefrontal cortex (Silvers et al., 2016). Since the publication of that article, a growing literature has highlighted a potential role for the NAcc in threat processing. For example, existing studies have implicated a NAcc-amygdala circuit that supports escape behavior among rats and adult humans (e.g., Ramirez et al., 2015; for reviews see Ledoux and Daw, 2018; Wong et al., 2022). We have extended this work to human adolescents, showing that functional connectivity between the NAcc and amygdala 1) becomes progressively more positive in response to escapable threats, 2) is higher in response to safety among adolescents with higher trait symptoms of anxiety and depression, and 3) increasingly differentiates threatening versus safe stimuli as adolescents get older (Rosenberg et al., 2024). Although institutionalization has been shown to alter NAcc recruitment in reward learning (Goff et al., 2013b, Mehta et al., 2010b), to our knowledge this work has not been extended to aversive learning paradigms.

### 1.5 The present study

The present study sought to test the hypothesis that ELA impairs threat and safety learning by altering coordination *between* the NAcc and amygdala during adolescence. We examined this in a unique sample of youth who were initially reared in orphanage care (PI) and a corresponding sample of comparison youth (Comp). We hypothesized the following (pre-registered after data collection at https://osf.io/txrg2): (1) PI group will be associated with poor threat and safety discrimination evidenced by NAcc-amygdala functional connectivity; (1a) This effect will be explained by elevated NAcc-amygdala functional connectivity for the non-reinforced (i.e., safe) escapable stimuli; (1b) If 1a is not supported, poor discrimination will alternatively be explained by diminished NAcc-amygdala functional connectivity for a reinforced (i.e., threatening) escapable stimulus (aligned with physiological evidence, e.g., Ruge et al., 2024; Klingelhöfer-Jens et al., 2025); (2) Extending our prior work, elevated NAcc-amygdala functional connectivity for the safe stimuli will be associated with internalizing symptoms across the full sample and moderated by PI status.

## 2 Materials and methods

### 2.1 Participants

Participants were 43 previously institutionalized (PI) adolescents internationally adopted from institutional orphanage care (ages 12.1–22.8 years, 31 female, mean age=17.23 years, SD=2.80 years) and 47 comparison (Comp) youth ages 9.9–22.9 years (24 female, mean age=15.23 years, SD=3.75 years). The Comparison youth, consisting of non-adopted youth who lived with their families throughout childhood, were recruited among the local population via flyers or from state birth records. The majority of PI youth were adopted during infancy and early childhood (among those reporting age of adoption, mean age=1.94 years, SD=1.61 years, range=0.7–96 months). Participants were recruited as part of a longitudinal study investigating the impact of early life experiences on the neural bases of socioemotional development. All research was approved by the University of California, Los Angeles (UCLA) Institutional Review Board and completed at UCLA. All adult participants provided consent, all minor participants provided informed assent, and all parents of minors provided informed consent.

Participant demographics are summarized in Table 1. Of note, *post hoc* tests found that the groups significantly differed in terms of age (t(86) = 2.59, *p* = .011), household income (>$100,000 vs Less; ꭓ^2^(1)= 6.60, *p* = .037), and intelligence (t(88) = -2.93, *p* = .004), such that PI youth tended to be older, tended to be in higher-income households, and tended to have lower intelligence scores.

**Table 1:** Participant demographics in the study.

	**PI Group (n = 43)**		**Comp Group (n = 47)**	
**Parent-Reported Participant Race**	**Total Number**	**Percent**	**Total Number**	**Percent**
African American or Black	0	0.00 %	7	14.89 %
American Indian or Alaska Native	0	0.00 %	0	0.00 %
Asian American	20	46.51 %	6	12.77 %
Caucasian	15	34.88 %	21	44.68 %
Native Hawaiian or Other Pacific Islander	0	0.00 %	1	2.13 %
Not Reported	2	4.65 %	3	6.38 %
Other	6	13.95 %	9	19.15 %
**Parent-Reported Participant Ethnicity**	**Total Number**	**Percent**	**Total Number**	**Percent**
Hispanic or Latino	0	0.00 %	5	10.64 %
Not Hispanic or Latino	35	81.40 %	42	89.36 %
Not Reported	8	18.60 %	0	0.00 %
**Parent-Reported Household Income**	**Total Number**	**Percent**	**Total Number**	**Percent**
Less than $10,000 per year	0	0.00 %	4	8.51 %
$10,001 - $25,000 per year	0	0.00 %	1	2.13 %
$25,001 - $40,000 per year	1	2.33 %	0	0.00 %
$40,001 - $55,000 per year	0	0.00 %	2	4.26 %
$55,001 - $70,000 per year	0	0.00 %	6	12.77 %
$70,001 - $40,000 per year	6	13.95 %	2	4.26 %
$85,001 - $100,000 per year	3	6.98 %	6	12.77 %
$100,001 - $150,000 per year	8	18.60 %	4	8.51 %
$150,001 - $200,000 per year	11	25.58 %	11	23.40 %
Greater than $200,000 pre year	7	16.28 %	9	19.15 %
Not Reported	7	16.28 %	2	4.26 %
**Parent-Reported Education (Self)**	**Total Number**	**Percent**	**Total Number**	**Percent**
Some High School	0	0.00 %	0	0.00 %
High School Degree	0	0.00 %	0	0.00 %
Some College	1	2.33 %	6	12.77 %
Community College / Two-Year Degree	2	4.65 %	1	2.13 %
Four-Year College Degree	15	34.88 %	7	14.89 %
Some Graduate School	2	4.65 %	4	8.51 %
Master's Degree	7	16.28 %	16	34.04 %
Doctoral Degree	4	9.30 %	9	19.15 %
Professional Degree	5	11.63 %	0	0.00 %
Other	0	0.00 %	2	4.26 %
Not Reported	7	16.28 %	2	4.26 %
**WASI-II Intelligence Score**	**Total Number**	**Percent**	**Total Number**	**Percent**
Less than 70	0	0.00 %	0	0.00 %
70–89	4	9.30 %	4	8.51 %
90–109	23	53.49 %	11	23.40 %
110–130	14	32.56 %	22	46.81 %
Greater than 130	2	4.65 %	10	21.28 %
**RCADS-P Symptom T-Score**	**Total Number**	**Percent**	**Total Number**	**Percent**
Less than 20	0	0.00 %	0	0.00 %
21–30	6	13.95 %	12	25.53 %
31–40	12	27.91 %	18	38.30 %
41–50	3	6.98 %	3	6.38 %
Greater than 50	3	6.98 %	1	2.13 %
Not Reported	19	44.19 %	13	27.66 %

Four subjects (two PI, two Comp) were excluded for excess head motion (as described below, this was established as participants with >20 % of volumes having average framewise displacement exceeding 0.9 mm or global BOLD signal changes above 5 standard deviations). Three subjects (one PI, two Comp) had incomplete task data and were included in multilevel models with incomplete task blocks included as missing data.

### 2.2 Aversive learning task

Participants completed an aversive learning task while inside an MRI scanner (described in detail elsewhere, see Silvers et al., 2016; Rosenberg et al., 2024). The task was organized into blocks based on three different stimulus types: a shape that was reinforced with an aversive sound (CS+_r_), the same shape without reinforcement (CS+_nr_), or a different shape that was never reinforced with an aversive sound (CS-). While viewing the stimuli, participants were instructed to respond with a button-press as soon as they saw the border of the shape thicken. Reinforcement of CS+_r_ with the aversive noise (unconditional stimulus; US) occurred at the same time that the border thickened. Participants were not informed of what would happen when they pressed the button. Critically, the button press terminated each trial regardless of stimulus type, and it also temporarily terminated the aversive noise during CS+_r_ blocks. Thus, the task modeled not only aversive learning but the ability to escape threat as well.

The US was a loud, metallic, high-frequency noise (Neumann et al., 2008). The US was titrated so that it was perceived as “annoying” but not painful before the task for each participant (maximum volume, 92 dB). This calibration has been previously used in studies of aversive learning (e.g., Rosenberg et al., 2024; Silvers et al., 2016). On CS+_r_ trials, participant responses co-terminated the US and CS+_r_ and another trial immediately began.

The task involved eight task blocks lasting approximately 27 s (three CS+_r_ blocks, three CS+_nr_ blocks, and two CS− blocks). The CS+ and CS- shapes were counterbalanced across participants. Every participant completed blocks in the same order: CS+_r1_, CS+_nr1_, CS-_1_, CS+_r2_, CS+_nr2_, CS-_2_, CS+_r3_, CS+_nr3_.

### 2.3 Symptoms of anxiety and depression

Of the recruited sample, n = 60 parents of the included participants (n = 24 PI, n = 36 Comp) completed the Revised Child Anxiety and Depression Scales (RCADS-P; Chorpita et al., 2000), a 47-item questionnaire evaluating symptoms of anxiety (e.g., “My child has trouble going to school in the mornings because of feeling nervous or afraid”) and depression (e.g., “My child feels worthless”). Responses range from 0 to 3 (0 = Never; 1 = Sometimes; 2 = Often; 3 = Always), with higher scores indicating more internalizing symptoms. To enhance clinical utility and interpretability, total scores were converted to T-scores.

### 2.4 FMRI data acquisition and analysis

#### 2.4.1 Acquisition

A 3 T Siemens Prisma scanner was used to acquire imaging data with a 32-channel head coil and a parallel (GRAPPA) system. A T1-weighted high-resolution MPRAGE image was acquired (TR = 2400 ms, TE = 2.22 ms, flip angle = 8°, FOV = 256 mm^2^, 0.8 mm^3^ isotropic voxels, 208 slices) for registration to functional runs. A T2* EPI BOLD sequence was used to acquire functional images (TR = 2000 ms, TE = 30 ms, flip angle = 75°, FOV = 192 mm^2^, 3 × 3 × 4 mm^3^ voxel resolution, 33 axial slices). A head-mounted mirror on the coil enabled participants to view an LCD back projector screen as they completed the aversive-learning task.

#### 2.4.2 Preprocessing

Functional images were inspected before processing to identify artifacts and biological abnormalities. No images warranted exclusion on the basis of obvious artifacts or biological abnormalities. For n = 3 subjects, the scan terminated prior to the completion of the full task. In these cases, data for the first seven out of eight task blocks were analyzed (i.e., not the third CS+_nr_ block). Analyses modeled these incomplete blocks as missing data (see *2.4.5 Functional Connectivity*).

We used the CONN functional connectivity toolbox v22a (Whitfield-Gabrieli and Nieto-Castanon, 2012),^1^ implementing the default preprocessing settings for volume-based analyses. The default procedure in SPM12 was used for realignment (Andersson et al., 2001) and slice-timing correction for interleaved acquisition (Henson et al., 1999). The Artifact Detection Tools (ART) software default settings (acquisitions with framewise displacement above 0.9 mm or global BOLD signal changes above 5 standard deviations) were used for the identification and censoring of outlier volumes default settings (acquisitions with global BOLD signal changes greater than 5 standard deviations or framewise displacement greater than 0.9 mm). Data were normalized to the Montreal Neurological Institute standard space (Friston et al., 1994) and resliced to 2 mm × 2 mm × 2 mm voxels. Data were smoothed using a 6 mm full-width at half-maximum Gaussian kernel.

#### 2.4.3 First-Level fMRI analyses

First-level covariates included eighteen motion regressors (*x*, *y*, and *z* displacement; pitch, roll, and yaw rotation; first- and second-level derivatives). Using anatomical component-based noise correction (Behzadi et al., 2007), we extracted the timeseries of activation from subject-specific white matter and cerebrospinal fluid masks and then applied principal components analysis to estimate physiological noise from these timeseries. We then included the resulting components as covariates in a denoising regression (see Whitfield-Gabrieli and Nieto-Castanon, 2012 for additional details). Finally, to remove low-frequency activity associated with scanner drift and additional high-frequency activity associated with physiological functioning, we applied a band-pass filter of 0.008–0.09 Hz.

#### 2.4.4 Task performance

Task-based reaction times (RTs) were calculated during each task block for each participant. We winsorized mean RTs for each task block, replacing RTs above the 95th percentile with RT values at the 95th percentile and replacing RTs below the 5th percentile with RT values at the 5th percentile.

We previously analyzed RTs among Comp youth, demonstrating a significant main effect of Block, such that RTs tended to get faster across task blocks, and a significant main effect of Stimulus, such that RTs for the CS+_r_ tended to be faster than RTs for the CS+_nr_ (Rosenberg et al., 2024). In the present study, we conducted multilevel modeling in Stata 18.0 to additionally test for a main effect Group, as well as a Group x Stimulus interaction. Models included random effects of the intercept and slope for each subject, fixed effects for Group, and fixed effects for Block and Stimulus as within-subject factors. We included Stimulus (i.e., CS+_r_, CS+_nr_, CS-) as a categorical variable in all analyses. Block was modeled as a categorical variable since the stimuli did not have an equal number of blocks (i.e., a continuous model would estimate RT values for a non-existent Block 3 of the CS-).

#### 2.4.5 Functional connectivity

The analysis strategy was identical to the approach taken in our prior study (Rosenberg et al., 2024). We extracted ROIs for the bilateral NAcc and bilateral amygdala using the Harvard-Oxford atlas (Desikan et al., 2006). Functional connectivity during the task was analyzed in the CONN Toolbox using generalized psychophysiological interaction (gPPI) (Friston et al., 1997). For each of the eight task blocks, we extracted beta weights for NAcc-amygdala connectivity and imported these values into Stata 18.0 for multilevel modeling. We first analyzed the Stimulus x Block interaction as a predictor of NAcc-amygdala connectivity (covarying for Group, Age, Sex, and Mean Framewise Displacement). We then analyzed the main effect of Group, Group x Stimulus interaction, and Group x Stimulus x Block interaction (covarying for Age, Sex, and Mean Framewise Displacement). All models included the intercept and slope as random effects for each subject and Group, Stimulus, and Block as fixed effects. Group and Stimulus were included as categorical variables in all analyses. Block was included as a categorical variable because the stimuli had an unequal numbers of blocks. To determine significance of the Stimulus x Block and Group x Stimulus x Block interactions, models with and without the interaction term were compared using the *lrtest* function in Stata. Individual blocks of task data were omitted from analysis if they yielded estimates of connectivity greater than 4 standard deviations from the mean for task blocks of the same stimulus type (n = 1; see Supplement).

#### 2.4.6 Association with internalizing symptoms

We first tested for a main effect of Group predicting RCADS-P (controlling for Age and Sex). We then used multilevel modeling to test for a main effect of RCADS-P, Group (PI, Comp) x RCADS-P interaction, and Group (PI, Comp) x Stimulus (CS+_r_, CS+_nr_, CS-) x RCADS-P interaction predicting NAcc-amygdala connectivity betas (covarying for Block, Sex, and Mean Framewise Displacement). Follow-up analyses evaluated Group x RCADS-P pairwise differences among the CS+_r_, CS+_nr_, and CS-.

#### 2.4.7 Exploratory analyses of age effects

As the ability to discriminate threat and safety tends to improve throughout adolescence (Jovanovic et al., 2014, Reinhard et al., 2022), we tested the main effect of Age, Group (PI, Comp) x Age interaction, and Group (PI, Comp) x Stimulus (CS+_r_, CS+_nr_, CS-) x Age interaction in predicting NAcc-amygdala connectivity betas (covarying for Block, Sex, and Mean Framewise Displacement). Follow-up analyses evaluated Group x Age pairwise differences among the CS+_r_, CS+_nr_, and CS-.

#### 2.4.8 Exploratory analyses of other neurocircuits

To explore differences in connectivity among other nodes in threat neurocircuitry, we extracted three additional ROIs (bilateral hippocampus, bilateral insular cortex, and dorsal anterior cingulate cortex) using the Harvard-Oxford Atlas and a fourth ROI (ventromedial prefrontal cortex) using a 5 mm sphere, based on coordinates from a prior meta-analysis (Fullana et al., 2016) as no ROI is included for this region in the Harvard-Oxford atlas. For each of the eight task blocks, we computed beta weights (covarying for Age, Sex, and Mean Framewise Displacement) of functional connectivity between the NAcc and each of these four target ROIs. We used a between-blocks omnibus contrast in the CONN Toolbox to identify significant pairwise effects between groups (*p*-unc <.05).

## 3 Results

We hypothesized that functional connectivity between the NAcc-amygdala would differ between the PI and Comp groups, such that the PI group would exhibit elevated connectivity for the safe CS+_nr_/CS-, or lower connectivity for the threatening CS+_r_. We additionally hypothesized that elevated NAcc-amygdala functional connectivity for the safe CS+_nr_/CS- would be associated with clinical symptoms, and that this relationship would be more pronounced among the PI versus Comp youth. Exploratory analyses evaluated Age as a predictor of NAcc-amygdala connectivity during the task. For each set of analyses, we explored pairwise group differences and emphasize that results should be interpreted as tentative given their exploratory nature of these effects. These analyses were not corrected for multiple comparisons, and it is important to see them as identifying leads for future follow-up work, rather than as definitive.

### 3.1 Reaction times

#### 3.1.1 Effects across the full sample

There was a significant main effect of Stimulus, such that RTs for the CS+_r_ tended to be faster than RTs for the CS+_nr_ (b_ CS+_nr_=18.82, b_CS-=5.93; ꭓ^2^(2)= 16.98, *p* < .001; see Fig. S1). There was also a significant Stimulus x Block interaction, such that RTs were faster for Block 3 of the CS+_r_ compared with Block 3 of the CS+_nr_ (b_ CS+_nr__Block2 =-2.90, b_ CS+_nr__Block3 =31.27, b_CS-_Block2 =16.66; ꭓ^2^(3)= 14.68, *p* = .002). These results collectively suggest participants learned to escape the CS+_r_ during the task.

#### 3.1.2 Associations with previous institutionalization

There was a marginally significant main effect of Group (b = −17.23, ꭓ2(1)= 3.06, *p* = .080), such that RTs tended to be slower among the PI versus Comp youth over and above the main effects of Stimulus and Block. There was not a significant Group x Stimulus (b_CS+_nr_=-3.22, b_CS-=-3.51; ꭓ^2^(2)= 0.16, *p* = .922) interaction in predicting RTs during the task. These results are broadly supportive of the notion that the PI and Comp participants exhibited comparable differentiation of the stimuli during the task (consistent with Silvers et al., 2016), but that PI participants may have responded more slowly throughout the task regardless of the stimulus. Considering possible group differences in IQ, follow-up analyses demonstrated that IQ was not a significant predictor of RT during the task (see Supplement).

### 3.2 NAcc-amygdala functional connectivity

#### 3.2.1 Effects across the full sample

**Fig. 1:**
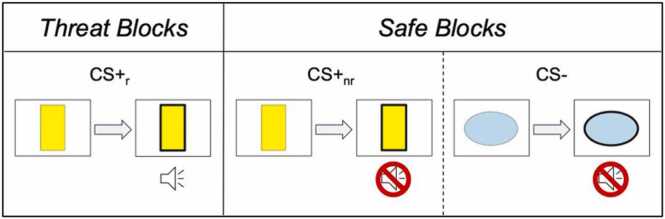
Design of the aversive learning task paradigm. During each trial, participants were instructed to press a button as soon as the border of the shape thickened. During CS+_r_ blocks, the border thickening coincided with an aversive noise, which terminated following the button-press. Participants completed eight total blocks (three CS+_r_, three CS+_nr_, two CS-).

The full model (Independent Variables: Group, Block, Stimulus, Block x Stimulus, Age, Sex, Mean Framewise Displacement) significantly explained variance in connectivity between the NAcc and amygdala (ꭓ^2^(11)= 21.55, *p* = .028). Extending previous work identifying a significant Stimulus x Block interaction among the Comp youth (Rosenberg et al., 2024), there was a significant Stimulus x Block interaction across the full sample (b_CS+_nr__Block2 =-.08, b_CS-_Block2 =-.21, b_CS+_nr__Block3 =-.35, ꭓ^2^(3)= 10.34, *p* = .016; Fig. 1).

#### 3.2.2 Associations with previous institutionalization

**Fig. 2:**
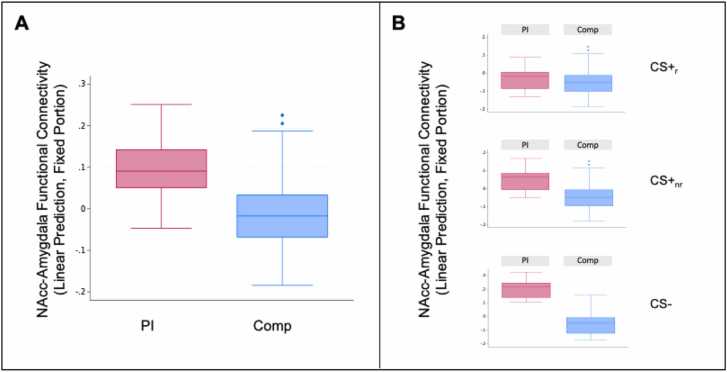
(A) Group differences in NAcc-amygdala functional connectivity during the aversive learning task. (B) Effects visualized for each stimulus type. PI youth showed greater NAcc-amygdala functional connectivity across the stimulus types and especially for the CS-.

There was a significant main effect of Group (b=-.14, ꭓ^2^(1)= 4.41, *p* = .036), such that the PI youth tended to show greater NAcc-amygdala connectivity during the task (Fig. 2A; for results including n = 1 outlier block, see Supplement). Contrary to hypotheses, there was not a significant Group x Stimulus interaction (b_CS+_nr_=-.07, b_CS-=-.23, ꭓ^2^(2)= 3.76, *p* = .152), suggesting that elevated connectivity among the PI youth did not differ by stimulus type (Fig. 2B). Nonetheless, as we hypothesized that the PI group would show elevated connectivity for either the CS+_nr_ or CS-, and as the Group x Stimulus interaction includes all three stimulus types, we explored pairwise group differences for each stimulus type. These exploratory analyses identified a significant pairwise group difference for the CS- (Z = -2.74, *p* = .006), such that the PI group showed greater connectivity for the CS- compared with the Comp group, but not the CS+_r_ (Z = -.63, *p* = .530) nor CS+_nr_ (Z = -1.46, *p* = .145). These results tentatively suggest that the main effect of Group may have been partially driven by CS- blocks.

**Fig. 3:**
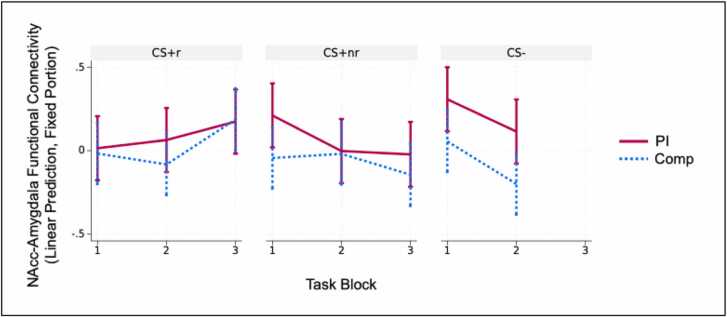
Group differences in NAcc-Amygdala functional connectivity depicted for each stimulus/block combination. Exploratory results tentatively found greater functional connectivity among the PI group during the second CS- block.

Furthermore, there was not a significant Group x Stimulus x Block interaction (CS+_r_ and Block 1 are reference conditions, b_CS+_nr__Block2 =.35, b_CS-_Block2 =.05, b_CS+_nr__Block3 =.09, ꭓ^2^(3)= 2.38, *p* = .497). Nonetheless, as we hypothesized that the PI group would show elevated connectivity for either the CS+_nr_ or CS- during the task, and as the three-way interaction compares across all blocks and stimulus types, we explored pairwise group differences for each stimulus/block pairing. These exploratory analyses identified a significant pairwise group difference for CS- Block 2 (Z = -2.30, *p* = .022), such that the PI group showed greater connectivity for CS- Block 2 compared with the Comp group (Fig. 3). Exploratory analyses also found marginally significant pairwise group differences for CS+_nr_ Block 1 (Z = -1.86, *p* = .063) and CS- Block 1 (Z = -1.84, *p* = .065). All other pairwise comparisons were non-significant (*p*’s > .28). Collectively, these results suggest the PI youth showed greater NAcc-amygdala connectivity throughout the task, with exploratory evidence tentatively suggesting effects were clearest for safe blocks.

### 3.3 Associations with trait anxiety and depression symptoms

#### 3.3.1 Association with group

Contrary to hypotheses, there was not a significant main effect of Group in predicting RCADS-P scores (t(54) = -1.18, *p* = .243).

#### 3.3.2 Association with NAcc-amygdala connectivity

Contrary to hypotheses, there was not a significant main effect of RCADS-P (b=.00, Z = .94, *p* = .348) nor a significant Group x RCADS-P interaction (b=.01, ꭓ^2^(1)= 1.62, *p* = .204) in predicting NAcc-amygdala connectivity. There was a marginally significant three-way Group x Stimulus x RCADS-P interaction (CS+_r_ is the reference condition, b_CS+_nr_=.00, b_CS-=.04, ꭓ^2^(2)= 4.73, *p* = .094), suggesting that the previously identified Stimulus x RCADS-P interaction among the Comp youth (Rosenberg et al., 2024) was not similarly evident within the PI group. Follow-up analyses of this effect identified a marginally significant Group x RCADS-P interaction specifically for the CS- (b=.04, ꭓ^2^(1)= 3.71, *p* = .054), but not the CS+_r_ (b=.00, ꭓ^2^(1)= .01, *p* = .938) nor CS+_nr_ (b=.00, ꭓ^2^(1)= .04, *p* = .838).

### 3.4 Exploratory analyses of age effects

#### 3.4.1 Reaction time

There was a significant main effect of Age (b=-5.55, Z = -3.28, *p* = .001), such that older participants tended to have faster RTs during the task. There was no Group x Age interaction (b=-1.29, Z = -.37, *p* = .713), Age x Stimulus interaction (CS+_r_ is the reference condition, b_CS+_nr_=1.46, b_CS-=2.23, ꭓ2(2)= 1.41, *p* = .494), or Group x Age x Stimulus interaction (ꭓ2(2)= 1.39, *p* = .500).

#### 3.4.2 Association with NAcc-amygdala connectivity

**Fig. 4:**
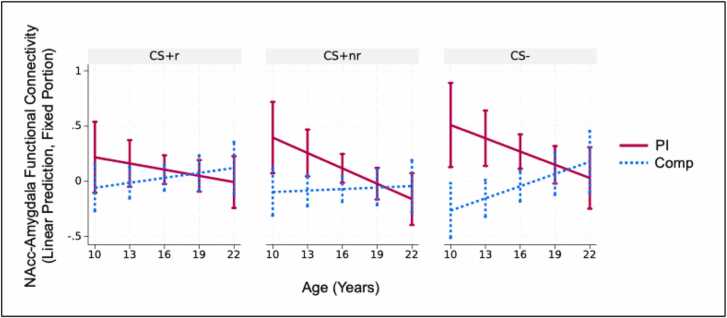
Group x Stimulus x Age interaction predicting NAcc-amygdala functional connectivity during the aversive learning task. Exploratory results tentatively suggest that functional connectivity for both the CS+_nr_ and CS- was elevated in the PI versus Comp group at younger but not older ages.

There was not a significant main effect of Age (b=.00, Z = -.37, *p* = .712). There was a significant Group x Age interaction (b=-.03, ꭓ^2^(1)= 6.82, *p* = .009), such that the PI group showed greater connectivity compared with the Comp group at younger but not older ages (Fig. 4). This cross-sectional age effect tentatively suggests that differences in threat-related NAcc-Amygdala connectivity among the PI group may diminish during adolescence. Exploratory analyses did not identify a significant Group x Age x Stimulus interaction (CS+_r_ is the reference condition, b_CS+_nr_=.02, b_CS-=.04, ꭓ^2^(2)= 1.35, *p* = .509). Exploratory follow-up analyses determined that the Group x Age interaction was significant for the CS+_nr_ (b=.06, ꭓ^2^(1)= 4.02, *p* = .045), such that among PI youth connectivity was negatively associated (i.e., became more similar to the Comp youth) with Age. The Group x Age interaction was not significant for the CS+_r_ (b=.04, ꭓ^2^(1)= 2.65, *p* = .104) nor the CS- (b=.06, ꭓ^2^(1)= 2.58, *p* = .108) (Fig. 4).

### 3.5 Exploratory analyses of other neurocircuits

No other NAcc-ROI pairs (bilateral hippocampus, bilateral insular cortex, dorsal anterior cingulate cortex, ventromedial prefrontal cortex) showed significant pairwise effects between groups during the task (all omnibus-contrast *p*’s > .05).

## 4 Discussion

This study evaluated if exposure to institutional orphanage care during early childhood, a profound form of ELA, alters functional connectivity between the NAcc and amygdala during aversive learning in adolescence. Prior work from our group has demonstrated that elevated functional connectivity of this circuit is associated with adaptive escape behavior in response to threats, and that elevated connectivity during safety is associated with trait internalizing symptoms in adolescence (Rosenberg et al., 2024). The present study extends those results by demonstrating that PI is associated with elevated NAcc-amygdala functional connectivity during the task. Exploratory analyses further demonstrated that PI-related elevations in NAcc-amygdala connectivity were evident during safe blocks and may diminish with increasing age. Results broadly suggest that PI alters neural mechanisms of threat/safety discrimination during early development, and that these differences may normalize throughout later stages of adolescence in adopted youth.

### 4.1 ELA and threat/safety discrimination

ELA is associated with altered threat detection, resulting in reduced discrimination between threats and safety (for a systematic review, see Ruge et al., 2024). Some evidence specifically suggests ELA is associated with elevated threat responding during safety learning, measured by physiological reactivity (Wolitzky-Taylor et al., 2014), as well as behavioral avoidance and threat-related brain activity (Marusak et al., 2021). Results from the present study suggest that PI youth specifically exhibit heightened neural responsiveness across stimulus types, here measured by NAcc-amygdala functional connectivity (which has been previously linked to adaptive escape behavior). Results tentatively suggested effects specific to the CS-, as PI youth on average tended to show positive NAcc-amygdala connectivity for the CS-, whereas Comp youth on average tended to show negative NAcc-amygdala connectivity for the CS-. In contrast, results from the present study did not provide evidence to support the hypothesis that ELA would be associated with decreased neural responsiveness to threats. Instead, the PI group showed significantly *higher* NAcc-amygdala connectivity throughout the task on average. Furthermore, although the PI youth tended to show slower RTs during the task, these results were not specific to the CS+_r_ and the PI youth responded faster for the CS+_r_ compared with the CS+_nr_ and CS-. Collectively, these results suggest that PI youth showed heightened threat responses across threatening and safe stimuli, including a change from negative to positive connectivity for the CS-, coinciding with slower RTs when discriminating threat versus safety.

### 4.2 ELA and threat neurodevelopment

Compared with typically-developing youth, it has been theorized that altered neurodevelopment among ELA-exposed youth reflects the absence of expected environmental features (e.g., supportive caregiving) (Nelson and Gabard-Durnam, 2020), prompting a prioritization of survival neurocircuitry over developmental needs (Callaghan and Tottenham, 2016). Results from the present study align with this theory, as ELA-exposed youth exhibited elevated NAcc-amygdala functional connectivity throughout the task. Considering that the NAcc-amygdala circuit plays a central role in adaptive responding to real threats (Ledoux and Daw, 2018, Ramirez et al., 2015, Rosenberg et al., 2024), elevations during the task may reflect a tendency for ELA-exposed youth to process all cues as potential threats. Alternatively, it could be that ELA alters neurodevelopment of the NAcc-amygdala circuit, such that the circuit is broadly responsive to threatening contexts (e.g., the aversive learning task) but is not specifically tuned to conditional stimuli within the threatening context. Regardless, considering that group differences in NAcc-amygdala functional connectivity were most apparent for the safe CS-, these results are broadly consistent with the suggestion that ELA is associated with high threat reactivity in non-threatening contexts.

### 4.3 ELA and psychopathology

ELA is associated with increased risk for psychopathology (Green et al., 2010b, Kessler et al., 2010, Macmillan et al., 2001, Norman et al., 2012), particularly during adolescence (McLaughlin et al., 2012). Of note, ELA is associated with alterations to threat and reward learning processes that are commonly targets of psychotherapy (e.g., exposure therapy, positive affect treatment) (McLaughlin, DeCross, et al., 2019). For example, poor discrimination of threat and safety cues during development can lead to overreliance on avoidance behaviors that increase the risk of developing anxiety disorders (Waters and Craske, 2016). PI youth tended to show elevated NAcc-amygdala connectivity during the task including the safe CS-; however, unlike Comp youth, internalizing symptoms did not explain this association further. One possibility is that altered NAcc-amygdala functional connectivity among the PI group reflects accelerated neurodevelopment of survival circuitry (Callaghan and Tottenham, 2016), such that adversity-exposed individuals broadly show reduced neural differentiation of threat and safety (consistent with Lecei and van Winkel, 2020; Saragosa-Harris et al., 2024). Elevated NAcc-amygdala functional connectivity may therefore be a common feature among PI youth irrespective of internalizing symptoms, whereas typically-developing youth may show greater variability in NAcc-amygdala functional connectivity according to individual differences in internalizing symptoms. An alternative explanation is that PI youth show reduced neural discrimination between threat and safety via alternative mechanisms (e.g., amygdala functional connectivity with the hippocampus or anterior cingulate cortex, see DeCross et al., 2022). Additional research is needed to evaluate these possibilities.

### 4.4 Reversing the detrimental effects of ELA

Although ELA may have profound impacts on neurodevelopment, it may be possible to reverse these effects to promote optimal developmental outcomes (McDermott et al., 2023). For example, PI youth in the present study were all adopted into stable homes, and the majority of these adoptions occurred during infancy and early childhood (nearly all were adopted before four years of age). Prior studies of PI have demonstrated that youth randomized to foster care (versus continued institutionalization) exhibit superior outcomes across a range of cognitive and socioemotional measures (Humphreys et al., 2015, Humphreys et al., 2022, Zeanah et al., 2017). Early intervention and facilitation of attentive caregiving may therefore provide ELA-exposed youth with necessary support to promote development beyond survival demands. Furthermore, as pubertal development coincides with a “recalibration” of stress reactivity among PI youth who have been adopted into supportive homes (Gunnar et al., 2019), adolescence may present a particularly opportune period of flexibility (Crone and Dahl, 2012) that enables threat-responsive neurocircuitry to normalize among PI youth. Early intervention may therefore aid in the *deceleration* of survival-related neurodevelopment during adolescence in ways that protect against risk for developing psychopathology (e.g., reducing threat responding to safe cues). Results from the present study are consistent with these perspectives, as exploratory analyses found that PI youth showed elevated NAcc-amygdala functional connectivity across stimulus types during early adolescence, but not during late adolescence and early adulthood. Additional research is needed to establish an evidence base for the precise timing and implementation of intervention strategies to promote optimal health outcomes among ELA-exposed youth.

### 4.5 Limitations and future directions

Several limitations should be considered when interpreting the results of the present study. First, although the task paradigm in this study was intentionally designed as a simple measure of aversive learning, ecologically valid paradigms involving common real-world scenarios (e.g., driving a car, social encounters) may offer unique insights into adolescent threat learning. Second, the PI and Comp groups comprised youth of differing age ranges (youngest PI=12.1 years; youngest Comp=9.9 years), such that the mean age of PI participants was greater than the Comp participants. It is possible that group differences in NAcc-amygdala connectivity may be partially attributable to age or pubertal effects, though we controlled for age. Several additional demographic differences between the PI and Comp youth may further limit interpretability (e.g., race, ethnicity). Third, the wide age range may introduce age-related heterogeneity into each sample, although the variance in age enabled us to explore cross-sectional age effects, highlighting potential avenues for future research. Fourth, as analysis of this task has previously been conducted among the Comp participants, analyses in the present study may be susceptible to Type I error and should be interpreted with caution. Fifth, as the task included fewer CS- blocks compared with the CS+_r_ and CS+_nr_, future research with a greater number of CS- trials may enhance inferences about safety learning. Sixth, as the present study was cross-sectional in nature, longitudinal research may provide necessary insights into how ELA alters trajectories of NAcc-amygdala functional connectivity over time, and if these changes coincide with emerging internalizing symptoms. Finally, this study did not specifically recruit youth on the basis of internalizing symptoms, and participant scores were largely in the sub-clinical range. Additional research is needed to examine if ELA is differentially associated with NAcc-amygdala connectivity among youth with and without psychopathology (e.g., anxiety disorders).

## 5 Conclusion

The present study evaluated if a history of institutionalization orphanage care is associated with differences in NAcc-amygdala functional connectivity. This circuit has previously been implicated in adaptive escape behaviors in response to real threats. We found that PI youth exhibited elevated NAcc-amygdala functional connectivity during an aversive learning task. Exploratory analyses demonstrated that differences between the PI and Comp youth were evident during safe blocks and most notable during early but not late adolescence, suggesting that PI-related differences in threat neurodevelopment may diminish as adolescents get older. Future research is needed to expand this work across a range of aversive learning paradigms and to evaluate longitudinal associations throughout adolescent development.

## Funding

This research was supported by the 10.13039/100000001National Science Foundation (NSF 1848004), the Hellman Fellow Award, and an APF Visionary Award (awarded to JAS). BMR was supported by the 10.13039/100000025National Institute of Mental Health under award number T32MH015750. JFGM, ASML, and NMSH were supported by the National Science Foundation Graduate Research Fellowship (DGE-1650604). JFGM, NMSH, and CFM were supported by a National Institute of Child Health and Human Development T32 Predoctoral Training Grant (T32HD091059). NMSH was supported by the UCLA Edwin W. Pauley Fellowship and the UCLA Eugene V. Cota-Robles Fellowship Grant. WJM and SMC were supported by funding from the UCLA Graduate Division. SMC was supported by the Lippman Fellowship. The content is solely the responsibility of the authors and does not necessarily represent the official views of the National Institutes of Health.

## CRediT authorship contribution statement

**Emilia Ninova:** Writing – review & editing, Data curation. **Yael Waizman:** Writing – review & editing, Data curation. **Benjamin M. Rosenberg:** Writing – review & editing, Writing – original draft, Project administration, Methodology, Formal analysis, Data curation, Conceptualization. **Silvers Jennifer:** Writing – review & editing, Writing – original draft, Supervision, Resources, Project administration, Methodology, Funding acquisition, Conceptualization. **Adriana S. Méndez Leal:** Writing – review & editing, Methodology, Data curation, Conceptualization. **João F. Guassi Moreira:** Writing – review & editing, Methodology, Data curation. **Elizabeth Gaines:** Writing – review & editing, Methodology, Data curation. **Natalie M. Saragosa-Harris:** Writing – review & editing, Methodology, Data curation. **Clare F. McCann:** Writing – review & editing, Methodology, Data curation. **Wesley J. Meredith:** Writing – review & editing, Methodology, Data curation. **Saché M. Coury:** Writing – review & editing, Methodology, Data curation.

## Declaration of Competing Interest

The authors declare that they have no known competing financial interests or personal relationships that could have appeared to influence the work reported in this paper.

## Data Availability

Data will be made available on request.

## Footnotes

1 https://web.conn-toolbox.org/fmri-methods/preprocessing-pipeline
