# Supplementary material for: Previous institutionalization is associated with elevated functional connectivity between the nucleus accumbens and amygdala during aversive learning
Source: Dev Cogn Neurosci. 2025 Sep 26;76:101617. doi: 10.1016/j.dcn.2025.101617 (PMC12513215; doi:10.1016/j.dcn.2025.101617)
Supplement: Supplementary file 1 — Supplementary material [file mmc1.docx]

**SUPPLEMENT**

***S1. Summary of Results in Main Text***

In the main text, we reported a significant main effect of Group (*p*=.036) such that the PI group showed greater NAcc-amygdala connectivity throughout the task. Follow-up analyses indicated that this effect was significant for the CS- (*p*=.006) and not significant for the CS+_nr_ (*p*=.145) nor the CS+_r_ (*p*=.530). Additional analyses reported a significant Group x Age interaction (*p*=.009) in predicting NAcc-amygdala functional connectivity.

***S2. Supplemental Analyses & Results***

Figure S1: Visual depiction of the RT distribution during the task, by Group.

*S2.1 RT Analyses*

We considered the possibility that group differences in cognitive functioning may inform potential group differences in RT during the task. Indeed, as noted in section 2.1, the groups showed significantly different IQ scores. Nonetheless, when including IQ in mixed models there was not a significant main effect of IQ in predicting RT during the task (b=-.25, Z=-1.32, *p*=.182).

*S2.2 NAcc-Amygdala Results*

Our preregistered analyses did not involve a plan to winsorize the NAcc-amygdala functional connectivity data. Nonetheless, we identified that one participant showed greater NAcc-amygdala connectivity during a single block of the task compared with other participants (4.15 standard deviations above the mean for this task block). The main text omits this block from analyses. To further understand the effects reported in the main text, we conducted supplemental analyses including this block winsorized to 1.96 standard deviations above the mean, resulting in the following:

1. The main effect of Group in predicting NAcc-amygdala functional connectivity **remained significant** (ꭓ2(1)=3.96, *p*=.047), such that the PI group showed greater functional connectivity throughout the task.
2. The pairwise Group comparison of NAcc-amygdala functional connectivity for the CS- **remained significant** (ꭓ2(1)=5.47, *p*=.019), such that the PI group showed significantly greater functional connectivity for the CS- during the task.
3. The Group x Age interaction in predicting NAcc-amygdala functional connectivity **remained significant** (ꭓ2(1)=6.95, *p*=.008). The simple Group x Age interaction **remained significant** for the CS+_nr_ (ꭓ2(1)=4.02, *p*=.045), such that among the PI youth connectivity for the CS+_nr_ was negatively associated (i.e., became more similar to the Comp youth) with age.

In sum, winsorization of NAcc-amygdala functional connectivity for the outlying task block did not change the significance of core analyses reported in the main text.
